# Biopromoters for Gas Hydrate Formation: A Mini Review of Current Status

**DOI:** 10.3389/fchem.2020.00514

**Published:** 2020-07-08

**Authors:** Yong-Tao Zhang, Fu-Lin Chen, Shi-Jie Yu, Fei Wang

**Affiliations:** ^1^College of Electromechanical Engineering, Shandong Engineering Laboratory for Preparation and Application of High-Performance Carbon-Materials, Qingdao University of Science & Technology, Qingdao, China; ^2^Military Representative Office of Army, Qingdao, China

**Keywords:** gas hydrate, biopromoter, kinetic promoter, biomass, mechanism

## Abstract

Gas hydrates have promising application prospects in the fields of future energy sources, natural gas storage and transportation, CO_2_ capture and sequestration, gas separation, and cold energy. However, the application of hydrate technologies is being restricted due to the slow formation rate of gas hydrates. Kinetic promoters have been receiving increased attention, given that they can improve the hydrate formation rate with very small doses and do not affect gas storage capacity. However, most kinetic promoters are non-renewable, petrochemical-derived, non-degradable materials, inevitably leading to resource waste and environmental pollution. Biopromoters, derived from biomass, are renewable, biodegradable, environmentally friendly, non-toxic (or low toxic), and economically feasible. This mini review summarizes the current status of already discovered biopromoters, including lignosulfonate, amino acid, biosurfactant, and biological porous structures, which have the potential to replace petrochemical-derived promoters in hydrate technologies. Finally, future research directions are given for the development of biopromoters.

## Introduction

Gas hydrates are a form of non-stoichiometric crystalline, in which water molecules form the host lattice via hydrogen bonds and guest gases are trapped in the host lattice via intermolecular forces (He et al., 2019). Gas hydrates have been getting increased attention due to their promising number of applications, such as in future energy sources, natural gas storage and transportation, CO_2_ capture and sequestration, gas separation, and cold energy (Sun and Kang, 2016; Veluswamy et al., 2018; He et al., 2019). However, the application of hydrate technology is restricted by the slow formation rate of hydrates.

Kinetic promoters can improve the hydrate formation rate with very small doses and do not affect gas storage capacity (He et al., 2019). Researchers have used various kinetic promoters for gas hydrate formation, such as synthetic surfactants, activated carbon, porous silica, metal nanoparticles, graphene, carbon nanotubes, glass beads, sand grains, and dry water (Siangsai et al., 2015; Chong et al., 2016; He et al., 2019). However, most of the promoters above are non-renewable, petrochemical-derived, non-degradable materials, which will inevitably lead to resource waste and environmental pollution. Synthetic surfactants show an obvious superiority compared with other promoters, particularly given the material cost, especially for sodium dodecyl sulfate (SDS) (He et al., 2019). However, synthetic surfactants, such as SDS, sodium tetradecyl sulfate (STS), and sodium hexadecyl sulfate (SHS) can cause chronic toxicity in living organisms (Lewis, 1991), which restricts the application of hydrate technologies. For example, Ocean CO_2_ sequestration in the form of CO_2_ hydrates is being considered as an effective way to decrease the CO_2_ content in the atmosphere (Sun and Kang, 2016). If CO_2_ hydrates sequestrated in ocean sediments are formed using synthetic surfactants with chronic toxicity, the ocean ecological environment will suffer a dramatically adverse impact once those promoters are leaked into the ocean.

Biopromoters derived from biomass are renewable, biodegradable, environmentally friendly, nontoxic (or low toxic), and economically feasible. Biopromoters could be considered as promising promoters instead of traditional promoters for the application of hydrate technology. With the purpose of achieving a comprehensive evaluation on the discovered biopromoters and creating effective guidance for future research, this mini review summarizes the promoting effects and promoting mechanisms of discovered biopromoters which have the potential to replace petrochemical-derived promoters in gas hydrate technologies.

## Biopromoter

In the last 10 years, scholars have explored and discovered some biopromoters that can be used for gas hydrate formation, which can be divided into four categories: (1) lignosulfonates (LSs), (2) amino acids, (3) biosurfactants, and (4) biological porous structures. These four categories of biopromoters will be separately discussed in the following sections. The promoting effects of different biopromoters under corresponding experimental conditions are listed in Table S1.

## LS

**Figure 1:**
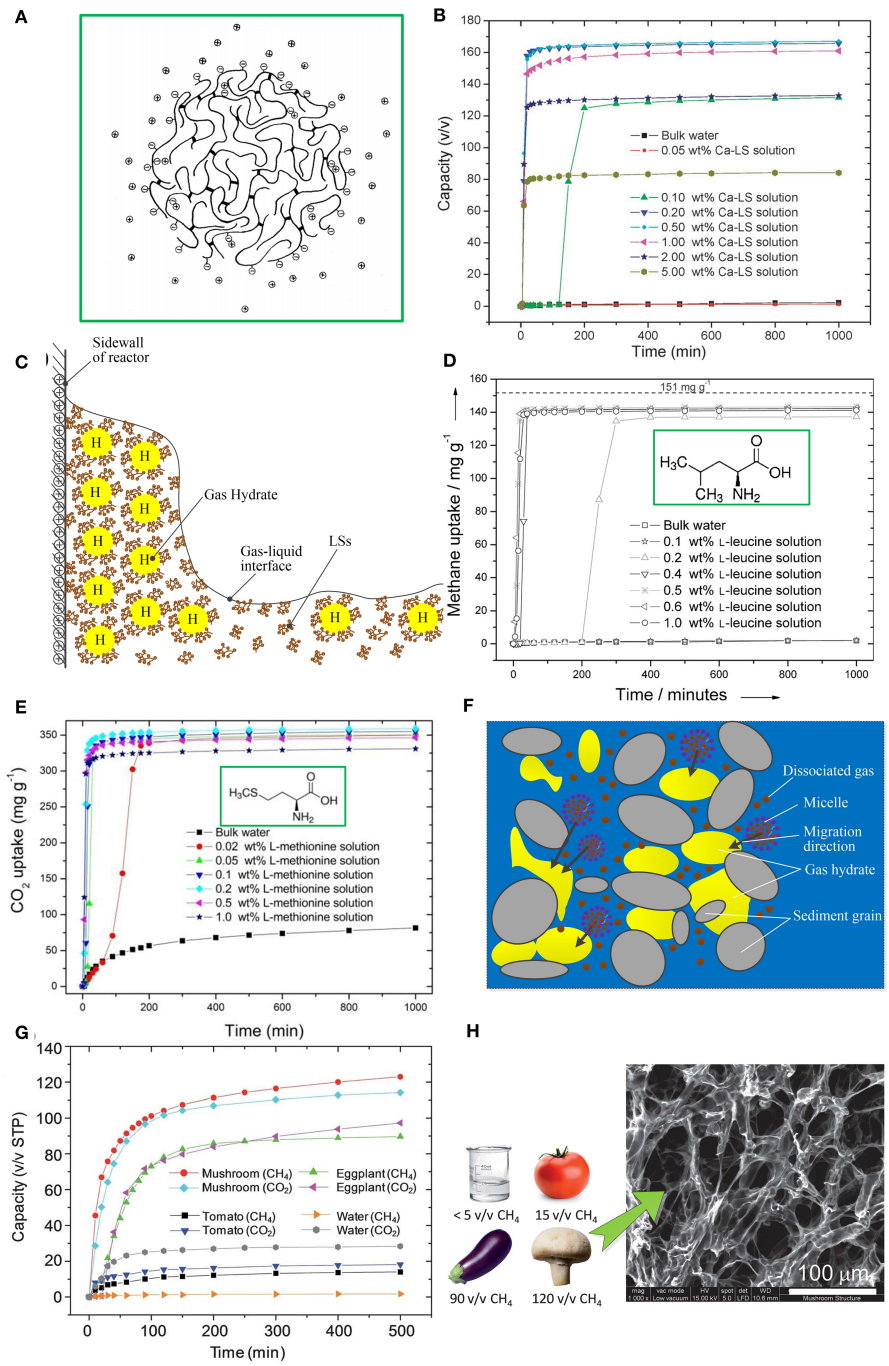
**(A)** Spherical microgel model of an LS macromolecule. Reproduced from Rezanowich and Goring (1960) with permission from Elsevier. **(B)** CH_4_ formation kinetics in a Ca-LS aqueous solution at 273.2 K and 9.5 MPa. Reproduced from Wang et al. (2012) with permission from the Royal Society of Chemistry. **(C)** Schematic diagram of the promoting mechanism of LSs. **(D)** CH_4_ formation kinetics in a L-leucine aqueous solution at 273 K and 9.5 MPa. Reproduced from Liu et al. (2015) with permission from John Wiley and Sons. **(E)** CO_2_ formation kinetics in L-methionine aqueous solution at 273.2 K and 3.3 MPa. Reproduced from Cai et al. (2017) with the permission from the John Wiley and Sons. **(F)** Schematic diagram of the promoting mechanism of biosurfactant in seabed sands/clay; **(G)** CH_4_ and CO_2_ formation kinetics for tomato, eggplant, and mushroom samples. Reproduced from Wang et al. (2013) with permission from the Royal Society of Chemistry. **(H)** SEM image of a mushroom sample showing a fine porous structure. Reproduced from Wang et al. (2013) with permission from the Royal Society of Chemistry.

LSs, as byproducts of the sulfite pulping process in the pulp and paper industry, are obtained by cutting α-*O*-4 ether bonds in nature lignin and sulphonating α- and/or γ-positions of the side chains of C9 units (Myrvold, 2008). The basic structure of LSs is a phenylpropane derivative, including a C3-C6 hydrophobic skeleton. LSs also contain hydrophilic groups, such as the sulfonic acid group, carboxyl group, and phenolic hydroxyl group. Although the microstructure of LSs in aqueous solutions is still inconclusive, it has been confirmed that LS macromolecules can generate cross-links and form spherical, disc-like, or sheet-like microgels with sulfonic acid groups located on its outer surface (Myrvold, 2008). Figure 1A shows a spherical microgel model of LS macromolecules (Rezanowich and Goring, 1960). The cross-link of LS macromolecules may offer a hydrophobic space for dissolving more guest gases, and sulfonic acid groups can associate cross-linked LS molecules with water molecules through a hydrogen bond (Wang et al., 2017).

Formation kinetics of gas hydrates in LS aqueous solutions were first reported by Wang et al. (2012). Figure 1B shows the formation kinetics of a CH_4_ hydrate in a calcium lignosulfonate (Ca-LS) aqueous solution with different concentrations (Wang et al., 2012). It can be seen that LS significantly improved the formation rate and gas storage capacity of the CH_4_ hydrate. The storage capacity of the CH_4_ hydrate reached 167 v/v (storage capacity was defined as the volume of guest gas stored in per unit volume of hydrate) within 1000 min in 0.5 wt% Ca-LS aqueous solution, *t*_90_ (the time to achieve 90% of the corresponding gas storage capacity) was only about 20 min, and the induction time reduced to <6 min. Among synthetic surfactants, the promoting effect of SDS is recognized as the best. However, a large amount of foam is generated during the dissociation process of the hydrate using SDS as a promoter, which not only influences the release of methane gas from the methane hydrate but also causes the loss of the SDS promoter in cyclic utilization (He et al., 2019). By contrast, the generated foam had a significant reduction during hydrate dissociation when the hydrate formed in a sodium lignosulfonate (Na-LS) aqueous solution, because the dissolution of Na-LS only generated a small amount foam (Mofrad et al., 2016). Besides, under certain concentrations, the CH_4_ storage capacity using Na-LS as a promoter is better than that of SDS, although the formation rate of Na-LS is still lower than that of SDS (Mofrad et al., 2016). For a CO_2_/CH_4_ gas mixture system, gas storage capacity under a Na-LS promoter was about 1.7 times higher than that in pure water, but the hydrate formation still had a longer induction time, ranging from 46.7 min to 400 min (Yi et al., 2019).

Most scholars think the promoting mechanism of LSs comes from their being capillarity-driven and their mass transfer. As observed by Wang et al. (2012), a CH_4_ hydrate grew upward along the inner wall of the reactor in an LS aqueous solution, which was a representative phenomenon from something that is capillarity-driven. As shown in Figure 1C, LS molecules can adsorb on the surface of forming hydrate particles under the action of hydrogen bonds between partial hydrophilic groups and hydrate molecules. Meanwhile, partial hydrophilic groups, such as sulfonic acid group and carboxyl group, are exposed to the outside surface of forming hydrate particles, which leads to mutual repulsion among forming hydrate particles under electrostatic action (Dicharry et al., 2016). And the hydrate particles become more wettable for water molecules due to hydrophilic groups. A water-wettable porous hydrate structure is formed, which drives the capillary action for sucking the water molecules to the reaction site of hydrate. CH_4_ hydrate formation has stronger capillarity-driven action than a CO2 hydrate formation (Daniel-David et al., 2015). On the other hand, the LS molecules arrange on the gas-liquid interface and reduce interfacial tension, which enhances the diffusion of gas molecules from a gas phase to a liquid phase. As a result, the supersaturation of gas molecules in a liquid phase promotes hydrate nucleation. However, it is necessary to use higher concentrations of LS to reduce interfacial tensions, compared with single molecule surfactants (Gupta and Washburn, 2014).

### Amino Acids

Amino acids are the basic constituent units of biologically macromolecular proteins and are an indispensable nutrient in the biological body. There are 20 species of amino acids obtained after proteolysis, around which researches have been focusing on both promoting hydrate formation and inhibiting hydrate formation in the last 10 years (Bavoh et al., 2019). The side chain of amino acids, ranging from a nonpolar alkyl chain to a charged or uncharged polar chain, plays a key role in their physico-chemical properties (Madeira et al., 2014).

Amino acids used for promoting gas hydrate formation were first reported by Liu et al. (2015). Leucine showed the best promoting effect for a CH_4_ hydrate among the surveyed Amino acids. As shown in Figure 1D, CH_4_ storage capacity reached 144 mg per g water (equivalent to a storage capacity of 161 v/v according to conversion Equation (1) in the Supplementary Material) and *t*_90_ was about 20 min. While for the CO_2_ system, L-methionine showed the best promoting effect, as shown in Figure 1E, where CO_2_ storage capacity reached 356 mg per g water (equivalent to a storage capacity of 144 v/v) and *t*_90_ was about 15 min (Cai et al., 2017). Summarizing previous studies, it can be found that for different guest gases the same amino acid will exert different effects. For example, L-histidine presented a promoting effect for a CH_4_ hydrate (Bhattacharjee et al., 2016), while presenting an inhibiting effect for a CO_2_ hydrate (Roosta et al., 2016). Leucine was reported to exert a poor promoting effect for an ethane hydrate and THF hydrate (Naeiji et al., 2014), but a favorable promoting effect for a CH_4_ hydrate (Liu et al., 2015). For a CH_4_ hydrate, the amino acids with a kinetic promoting effect are listed in order at concentration of 0.5 wt% as follows: L-leucine > L-isoleucine > D-leucine > L-methionine > L-phenylalanine > L-tryptophan > L-valine > L-arginine > L-glutamic acid > L-histidine > L-threonine (Liu et al., 2015). For CO_2_ hydrate, the amino acids with a kinetic promoting effect are listed in order as follows: L-methionine > L-norleucine > L-tryptophan > L-norvaline > n-hexylamine at concentration of 0.2 wt% reported by Cai et al. (2017) and L-methionine > L-cysteine > L-valine > L-threonine > L-phenylalanine at concentration of 0.5 wt%, reported by Prasad and Kiran (2018).

Most authors think the promoting effect comes from the surface activity of amino acids generated by the amine group, carboxylic group, and side chain. Given that the amine group and carboxylic group have hydrophilic properties, the amino acids with an aromatic sided chain and hydrophobic properties generally present a promoting effect (Bavoh et al., 2019). The formation of a hydrate film on the gas-liquid interface at the initial formation stage reduces the diffusion of gas molecules from a gas phase to a liquid phase. The surface activity of amino acids can restrain the formation of this initial hydrate film, which enhances gas mass transfer. The forming hydrates in an amino acid aqueous solution are very flexible and expandable, which indicates that being capillarity-driven also plays a role in the growth phase of hydrates (Veluswamy et al., 2016). In addition, amino acids can chemically adsorb CO_2_ molecules in a CO_2_ system via a zwitterionic mechanism, which is speculated to influence CO_2_ hydrate formation (Zhang et al., 2018). The CO_2_ molecule firstly reacts with the amine group, producing a zwitterion, and then the zwitterion reacts with the amine group, producing amino acid salt. The CO_2_ adsorption rate is related to the promoting effect of CO_2_ hydrate formation (Bavoh et al., 2019).

### Biosurfactant

Biosurfactants are secreted metabolites with surface activity during the metabolism of microorganisms under certain conditions. Microorganisms such as *Pseudomonas aeruginosa* and *Bacillus subtilis* were found from exploiting natural gas hydrate samples in the Gulf of Mexico (Lanoil et al., 2001). These two microorganisms can produce biosurfactants, i.e., rhamnolipid and surfactin, respectively, which has attracted the attention of hydrate researchers. Rhamnolipid is composed of 1-2 rhamnose rings (hydrophilic group) and saturated or unsaturated hydroxy fatty acids (hydrophobic group). Surfactin is a combination of a peptide ring containing 7 amino acid residues and β-hydroxy fatty acid containing 13-16 carbons by lactone bond (Arora et al., 2014).

Surfactin and rhamnolipid were first proven to have a promoting effect for natural gas hydrate formation in seawater-saturated sand/clay by Rogers et al. (2003), in which the hydrate formation rate increased by 96–288% and induction time decreased by 20–71%. Rhamnolipid was used as a co-promoter in a C-type silica-gel bed for promoting CH_4_ hydrate formation (Arora et al., 2016). As a result, the hydrate formation rate increased by 42.97% and induction time decreased by 22.63%, compared with a C-type silica-gel bed with saturated pure water. Biosurfactants also exhibited comparability with SDS. According to the research of Jadav et al. (2017), for 200 ppm surfactin, rhamnolipid, and SDS aqueous solution, the conversion rate from CH_4_ to hydrate was 42.7, 47.3, and 33.3%, and induction time was about 0.21, 0.23, and 1.13 h, respectively.

Biosurfactants have a lower critical micellar concentration (CMC) compared with synthetic surfactants (Arora et al., 2014). The CMC of the rhamnolipid seawater solution was about 13 ppm, which was easily achieved through minimal microbial activity in seabed sands/clay (Rogers et al., 2003). As shown in Figure 1F, the promoting effect of biosurfactants in seabed sands/clay was thought to be due to a micelle migration process, where micelles, dissolving hydrocarbon gas, migrated through seabed sand/clay with saturated seawater to the hydrate formation zone. On the other hand, both surfactin and rhamnolipid belong to anionic surfactants. Surfactin and rhamnolipid molecules can adsorb on the surface of hydrate particles to form a loose hydrate structure and enhance capillarity-driven action (Jadav et al., 2017). Rhamnolipid presents a better promoting effect compared with surfactin. This may be due to the difference in molecular structure. The anionic groups in surfactin and rhamnolipid molecules are nitrogen bonding and carboxylate, respectively, while rhamnolipid has more tails than surfactin, which helps to enhance the adsorption of rhamnolipid molecules on the surface of hydrate particles (Jadav et al., 2017). Rhamnolipid with a concentration of not <0.5 wt% was proved to form well-dispersed CH_4_ hydrate morphology in an oil-water system (Hou et al., 2018), which has an anti-agglomeration function. Besides, surfactin and rhamnolipid can also enhance mass transfer between the gas phase and liquid phase (Arora et al., 2014).

### Biological Porous Structure

Various biological structures have evolved for tackling gas transport and enhancing mass transfer in nature, such as alveoli, gills, stoma of leaves, etc., which encourages scholars to find some natural biological structures for promoting gas hydrate formation. Wang et al. (2013) first studied the promoting effect of the biological porous structure from mushroom, eggplant, and tomato on CH_4_ and CO_2_ hydrate formation, as shown in Figure 1G. CH_4_ storage capacity could reach 120 and 90 v/v within 500 min in mushroom and eggplant samples, respectively. CO_2_ storage capacity was similar to CH_4_ storage capacity. The better promoting effect benefits from the large surface-to-volume ratio and fine porous structure, as shown in Figure 1H, which improves gas mass transfer and helps to form loose gas hydrates with a biological porous structure as a framework. However, when mushroom and eggplant samples were used for the second hydrate cycle, there was a significant drop in gas storage capacity and formation rate. This was because the porous structure was destroyed after a hydrate-decomposition process.

Given that natural biological porous materials lack the structural stability for use in the recycling application of promoters in hydrate technologies, artificially biological structures may provide a better recycling stability. Nambiar et al. (2015) applied a porous cellulose foam fixed-bed for a hydrate-based CO_2_ separation from a CO_2_/H_2_/C_3_H_8_ gas mixture. They found that, compared with the saturation level of 100%, a cellulose foam fixed-bed under a saturation level of 50% presented a better promoting effect, because there was more available gas for gas mass transfer and migration of water molecules in the porous structure during hydrate formation. Unfortunately, there was no report on the recycling performance of a cellulose foam fixed-bed in the paper.

## Conclusion and Prospect

This mini review summarizes the current status of already discovered biopromoters, including LSs, amino acids, biosurfactants, and biological porous structures. In general, the order of promoting effect from strong to weak is as follows: LSs > amino acids > biological porous structures > biosurfactants. The surface activity and capillarity-driven action of LSs, amino acids, and biosurfactants play key roles in promoting gas hydrate formation. A biological porous structure improves gas mass transfer and helps to form loose gas hydrates. The following research direction should earn more attention in the future:

1. There is no consensus on the promoting mechanisms of biopromoters. Further study on promoting mechanisms is necessary, which would help to provide guidance for the selection of biologic materials as biopromoters.
2. At present, biopromoters have not shown enough advantages to replace petrochemical-derived promoters in gas hydrate technologies, particularly given the promoting effect. The gas storage capacity under biopromoters can be better than that under petrochemical-derived promoters, but only at certain concentrations. However, there is still a gap between the formation rates of gas hydrate under biopromoters and petrochemical-derived promoters. Nature is a treasury of biologic materials. It is necessary to seek new promising natural biopromoters to achieve a higher formation rate.
3. Artificially preparing biopromoters through chemical modification or by constructing a porous structure could be a promising approach for improving the promoting effect and recycling performance of promoters in hydrate technologies.

## Author Contributions

FW conceived the structure of the manuscript. F-LC collected materials and data. Y-TZ wrote the manuscript. S-JY wrote the Supplementary Material. FW revised and approved the manuscript.

## Conflict of Interest

The authors declare that the research was conducted in the absence of any commercial or financial relationships that could be construed as a potential conflict of interest.
