# Supplementary material for: Biopromoters for Gas Hydrate Formation: A Mini Review of Current Status
Source: Front Chem. 2020 Jul 8;8:514. doi: 10.3389/fchem.2020.00514 (PMC7360788; doi:10.3389/fchem.2020.00514)
Supplement: Supplementary file 1 [file Data_Sheet_1.PDF]

Biopromoters for Gas Hydrate Formation: A Mini Review of Mechanism and Current Status

Yong-Tao Zhang[a], Fu-Lin Chen[a], Shi-Jie Yu[b], Fei Wang[a*]

[a]College of Electromechanical Engineering, Shandong Engineering Laboratory for Preparation and Application of High-performance Carbon-materials, Qingdao University of Science & Technology, Qingdao 266061, China.

[b]Military Representative Office of Army, Qingdao 266061, China.

[*]Corresponding author: elliot_wang@qust.edu.cn

TABLE S1 List of some representative studies using biopromoters for promoting gas hydrate formation

No.	Promoter	Gas system	Formation condition	Promoting effect	Ref.
1	Ca-LS	CH_4	Temperature: 273.2 K; Initial pressure: 9.5 MPa; Without stirring.	CH_4 storage capacity reached 167 v/v in 1000 min, t_{90}[a] was about 20 min, and induction time was about 6 min.	Wang et al., 2012
2	Na-LS	CH_4		CH_4 storage capacity reached 170 v/v in 1000 min, t_{90}[a] was about 30 min, and induction time was about 8 min.	
3	K-LS	CH_4		The promoting effect of K-LS was slightly less than that of Na-LS.	
4	Na-LS	CH_4	Temperature: 275.15 K; Initial pressure: 10.9 MPa; Stirring rate: 200 r/min.	Under the concentration of 0.05 wt%, CH_4 storage capacity using Na-LS as promoter was better than that of SDS; The stability of CH_4 hydrate using Na-LS as promoter was better than that of SDS; In the dissociation process of the hydrate formed in Na-LS aqueous solution generated less foam, compared with SDS.	Mofrad et al., 2016

TABLE S1 (*continued*)

No.	Promoter	Gas system	Formation condition	Promoting effect	Ref.
5	Na-LS	$CH_4 + CO_2$	Temperature: 277.15 K; Initial pressure: 5.2 MPa; Stirring rate: 150 r/min.	The gas hydrate formation had longer induction time (from 46.7 min to 400 min), but the gas storage capacity was about 1.7 times higher than that in pure water.	Yi et al., 2019
6	Na-LS + THF	$CH_4 + CO_2$	Temperature: 277.15 K; Initial pressure: 2.8 MPa; Stirring rate: 150 r/min.	The gas hydrate formation had no induction time, but the gas storage capacity was lower than that in pure water.	
7	L-leucine	CH_4 and natural gas	Temperature: 273 K; Initial pressure: 9.5 MPa; Without stirring.	CH_4 storage capacity reached 144 mg per g water (161 v/v) in 1000 min, t_{90}[a] was about 20 min; Generation of foam during hydrate decomposition was avoided.	Liu et al., 2015
8	L-isoleucine > D-leucine > L-methionine > L-phenylalanine > L-tryptophan			Promoting effect was close to L-leucine and decreased progressively.	
9	L-valine			CH_4 storage capacity reached 138 mg per g water (154 v/v) in 1000 min.	
10	L-threonine			CH_4 storage capacity reached 80 mg per g water (89 v/v) in 1000 min.	
11	L-alanine			No apparent promoting effect.	
12	L-methionine	CO_2	Temperature: 273.2 K; Initial pressure: 3.3 MPa; Without stirring.	CO_2 storage capacity reached 356 mg per g water (144 v/v) in 1000; t_{90}[a] was about 15 min.	Cai et al., 2017

TABLE S1 (*continued*)

No.	Promoter	Gas system	Formation condition	Promoting effect	Ref.
13	L-norleucine			CO_2 storage capacity reached 362 mg per g water (147 v/v) in 1000; t_{90}[a] was about 28 min.	
14	L-tryptophan			CO_2 storage capacity reached 356 mg per g water (144 v/v) in 1000; t_{90}[a] was about 26 min.	
15	L-norvaline			CO_2 storage capacity reached 361 mg per g water (146 v/v) in 1000; t_{90}[a] was about 400 min.	
16	n-hexylamine			CO_2 storage capacity reached 135 mg per g water (55 v/v) in 1000.	
17	n-hexanoic			CO_2 storage capacity reached 56 mg per g water (23 v/v) in 1000.	
18	L-methionine	CO_2	Cooling the reactor from 298 K under 3.3 MPa; Stirring rate: 0 r/min and 300 r/min.	CO_2 storage capacity reached about 60 mmol and 70 mmol per mol water under stirring of 0 r/min and 300 r/min, respectively.	Prasad and Kiran, 2018
19	L-cysteine			CO_2 storage capacity reached about 60 mmol and 57 mmol per mol water under stirring of 0 r/min and 300 r/min, respectively.	
20	L-valine			CO_2 storage capacity reached about 61 mmol and 62 mmol per mol water under stirring of 0 r/min and 300 r/min, respectively.	
21	L-threonine			CO_2 storage capacity reached about 48 mmol and 12 mmol per mol water under stirring of 0 r/min and 300 r/min, respectively.	

TABLE S1 (*continued*)

No.	Promoter	Gas system	Formation condition	Promoting effect	Ref.
22	L-phenylalanine			CO_2 storage capacity reached about 53 mmol and 19 mmol per mol water under stirring of 0 r/min and 300 r/min, respectively.	
23	Surfactin	CH_4	Temperature: 273.15K; Initial pressure: 7 MPa; Stirring rate: 400 r/min.	Conversion rate from CH_4 to hydrate reached 42.7%; Induction time was about 0.21 h.	Jadav et al., 2017
24	Rhamnolipid	CH_4		Conversion rate from CH_4 to hydrate reached 47.3%; Induction time was about 0.23 h.	
25	Mushroom bioclathrate	CH_4 or CO_2	Temperature: 273.2 K; Initial pressure: 8.7 MPa; Without stirring.	CH4 storage capacity reached 123 v/v in 500 min; CO2 storage capacity was similar to CH4 storage capacity.	Wang et al., 2013
26	Eggplant bioclathrate	CH_4 or CO_2		CH4 storage capacity reached 95 v/v in 500 min; CO2 storage capacity was similar to CH4 storage capacity.	
27	Tomato bioclathrate	CH_4 or CO_2		The promoting effect was poor.	
28	Cellulose foam fixed-bed	$CO_2 + H_2 + C_3H_8$	Temperature: 274.2 K; Initial pressure: 6 MPa; Without stirring.	Average induction times of cellulose foam fixed-bed with saturation levels of 100% and 50% were 1.83 min and 0.55 min, respectively; Gas uptake reached near 0.07 mol gas per mol water for cellulose foam fixed-bed with saturation levels of 50%.	Nambiar et al., 2015

[a] the time to achieve 90% of corresponding gas storage capacity.

Storage Capacity Conversion Equation

There are different evaluation standards for storage capacity of gas hydrate in different literatures. The storage capacity are defined as the volume of guest gas stored in per unit volume of hydrate (v/v) in most literatures, while there are also some literatures which define storage capacity as the quality of guest gas stored in per unit quality of water (mg/g). In order to provide a uniform standard for discussing the promoting effect, the storage capacity under evaluation method of mg/g is converted to evaluation method of v/v using equation (1):

$$C_{\mathrm{v/v}} = \begin{cases} \dfrac{M_{\mathrm{H_2O}}}{M_{\mathrm{CH_4}}} \dfrac{1000V_{mg}}{V_{mw}+\Delta V} C_{\mathrm{mg/g}} & \text{For CH}_4\text{hydrate} \\ \dfrac{M_{\mathrm{H_2O}}}{M_{\mathrm{CO_2}}} \dfrac{1000V_{mg}}{V_{mw}+\Delta V} C_{\mathrm{mg/g}} & \text{For CO}_2\text{hydrate} \end{cases} \tag{1}$$

where $C_{\mathrm{v/v}}$ and $C_{\mathrm{mg/g}}$ are storage capacities under evaluation method of v/v and mg/g, respectively; $M_{\mathrm{H_2O}}$, $M_{\mathrm{CH_4}}$ and $M_{\mathrm{CO_2}}$ are relative molecular masses of H_2O, CH_4 and CO_2, respectively; V_{mg} and V_{mw} are the molar volumes of gas and water under standard temperature and pressure, respectively; ΔV is the molar volume difference between hydrate and water.
